# Immunomodulatory regulator blockade in a viral exacerbation model of severe asthma

**DOI:** 10.3389/fimmu.2022.973673

**Published:** 2022-11-21

**Authors:** Ben Nicholas, Hyun-Hee Lee, Jane Guo, Milenko Cicmil, Cornelia Blume, René De Waal Malefyt, Ratko Djukanović

**Affiliations:** ^1^ Division of Clinical and Experimental Sciences, University of Southampton Faculty of Medicine, Southampton General Hospital, Hampshire, United Kingdom; ^2^ Oncology & Immunology Discovery, Merck Research Laboratories, Boston, MA, United States; ^3^ Merck Research Laboratories, Palo Alto, CA, United States

**Keywords:** asthma, allergy, immuno-modulation, viral, exacerbation

## Abstract

Asthmatics are more susceptible to viral infections than healthy individuals and are known to have impaired innate anti-viral defences. Influenza A virus causes significant morbidity and mortality in this population. Immuno-modulatory regulators (IMRs) such as PD-1 are activated on T cells following viral infection as part of normal T cell activation responses, and then subside, but remain elevated in cases of chronic exposure to virus, indicative of T cell exhaustion rather than activation. There is evidence that checkpoint inhibition can enhance anti-viral responses during acute exposure to virus through enhancement of CD8+T cell function. Although elevated PD-1 expression has been described in pulmonary tissues in other chronic lung diseases, the role of IMRs in asthma has been relatively unexplored as the basis for immune dysfunction. We first assessed IMR expression in the peripheral circulation and then quantified changes in IMR expression in lung tissue in response to ex-vivo influenza infection. We found that the PD-1 family members are not significantly altered in the peripheral circulation in individuals with severe asthma but are elevated in pulmonary tissues following ex-vivo influenza infection. We then applied PD-1 Mab inhibitor treatment to bronchial biopsy tissues infected with influenza virus and found that PD-1 inhibition was ineffective in asthmatics, but actually increased infection rates in healthy controls. This study, therefore, suggests that PD-1 therapy would not produce harmful side-effects when applied in people with severe asthma, but could have important, as yet undescribed, negative effects on anti-viral responses in healthy individuals that warrant further investigation.

## Introduction

PD-1 is an immuno-inhibitory receptor belonging to the CD28 family which is predominantly expressed on T cells acutely activated by exposure to specific antigens (1). Engagement of PD-1 by its ligands, PD-L1 and PD-L2, suppresses effector T cell function through deletion/apoptosis, inhibition of proliferation, and/or production of cytokines such as IL-2 and IFN-γ. The PD-L1 ligand is expressed on haematopoietic cells and tissues such as the small intestine and placenta, while PD-L2 is expressed mainly on macrophages and dendritic cells. Both of the PD1 ligands are upregulated by cell activation. PD-L1 expression is key to effector T cell regulation during disease pathogenesis; thus, tumour cells expressing PD-L1 use the PD-1-PD-L1 pathway as a mechanism to evade recognition/destruction by the immune system by suppressing effector T cell killing of tumour cells.

Blockade of PD-1 or PD-L1 to inhibit T cell effector suppression by ligand engagement is increasingly used in the context of anti-tumour immunotherapy where it is effective against tumours with demonstrable PD-L1 expression, but its effectiveness in infectious disease has not been well explored (2, 3). Chronic antigen stimulation of the PD-1 pathway can lead to T cell exhaustion (4), accompanied by PD-1 elevation. This is a feature of viral infections, such as HIV and HBV, and in chronic inflammatory conditions, such as chronic obstructive pulmonary disease (COPD), and has been observed during acute exposure to influenza virus (5), although a causal link has not been proven. Little information is available on the expression and/or modulation of the PD-1 family in the context of chronic lung diseases, however there is some evidence that PD-1 blockade might assist with viral clearance in HIV by reducing latency (6). PD-1 blockade enhances CD8 cytokines such as IFN-γ (7) and is only partially effective in enhancing CD4 T cell cytokine production in counteracting HBV infection (8).

The aim of the current study was to assess the expression of PD-1 and its ligands in patients with asthma and to determine whether PD-1 blockade can influence T cell responses to influenza virus infection of the lungs and, thereby, serve as a treatment for virus-induced asthma exacerbations. Influenza virus infections cause significant morbidity and mortality globally, especially in patients with pre-existing chronic respiratory conditions such as asthma and COPD, but the exact molecular basis for increased susceptibility in people with chronic respiratory conditions is unclear. Using a bronchial explant model in which intracellular virus was quantified by flow cytometry using anti-viral nucleoprotein (NP) antibody, we have previously shown that corticosteroid treatment in healthy individuals is likely to increase susceptibility to viral infection by suppressing innate anti-viral pathways, an effect that is not seen in asthmatics. The human tissue explant model of viral infection maintains tissue-architecture during the infection process, including the presence of multiple inflammatory cell types, allowing the effect of asthma and viral infection to be compared in multiple resident cell types simultaneously. This model has been demonstrated to reflect human *in-vivo* viral tropism, influenza virus T cell epitope presentation, and inflammatory mediator release patterns more effectively than *in-vitro* cell systems, and we have previously demonstrated using lung parenchyma explants that PD-L1 expression patterns post-influenza infection are modulated in chronic obstructive pulmonary disease (5, 9)

Studies by others have suggested abnormal expression of the PD-1 pathway in atopic individuals and allergic asthma (10, 11) but have not established whether anti-PD-1 therapy could be useful or detrimental in the treatment of allergic asthmatics in the context of acute viral exposure.

In this study, we first assessed the *in-vivo* expression of a number of immunomodulatory receptors (IMRs) on circulating peripheral blood mononuclear cells (PBMC). Because of the potential risk of *in vivo* infection of patients, the second, mechanistic, part of the study was conducted in an established bronchial explant model in which bronchial biopsies were infected *ex vivo* where the interactions between PD-1 and its ligand could be safely inhibited.

## Materials and clinical samples

### Materials

Influenza virus A/H3N2/X31 and A/H3N2/Wisconsin/67/2005 seed stocks were from the National Institute for Biological Standards and Control (NIBSC), UK, propagated in embryonated SPF-free chicken eggs and purified from egg allantoic fluid by sucrose density gradient ultracentrifugation (Virapur LLC, San Diego, USA). Stock viral titre was determined by MDCK plaque assay using standard protocols. Monoclonal anti-influenza NP antibody conjugated to FITC was from BD (Cowley, Oxford, UK). PD-1 blocking antibody was a humanised IgG4κ Mab containing the S228P mutation (Genscript 1B8/HuPD1B-3 (59AGY). The control blocking antibody was a humanised anti-RSV IgG4κ Mab (S228P) (60AGK).

### Peripheral lung tissue samples

Resected human lung tissue was obtained from patients undergoing surgical lobectomy to remove lung cancer at Southampton University Hospital. Parenchymal tissue from donors without evidence of gross abnormalities, distant from the resection margin and any gross pathology, tissue was dissected and used within 2 h.

### Bronchial lung tissue samples

Ten healthy and 10 severe allergic asthmatics, aged between 18 and 65, provided written consent and volunteered for the study (**Table 1**). Specific inclusion criteria for the healthy participants included normal lung function tests (spirometry and diffusing capacity for carbon monoxide [DLCO]), while the asthmatics were required to be on Step 4 of asthma management as per BTS/SIGN asthma guidelines (12) and to be atopic as judged by positive skin tests to at least one common aero-allergen. The asthmatics were all on regular treatment with high-dose inhaled corticosteroids (ICS) and long-acting beta agonists (LABA); they required a minimum of three puffs pf salbutamol per week to relieve acute asthma symptoms, which defined them as not fully controlled. A minimum of 4 weeks since the last acute exacerbation was a requirement prior to sputum and bronchoscopy visits. Exclusion criteria in both groups included smoking during the past 12 months and, in the asthmatics, any evidence of overlap with COPD. If there was a smoking history of >10 pack years, then asthma diagnosis needed to have been made before the age of 40 and there had to be objective evidence of reversibility of forced expiratory volume in one 1 second (FEV_1_)>12% and 200ml.

**Table 1:** Clinical characteristics of healthy volunteers and severe allergic asthmatics recruited to study for the ex-vivo infection of bronchial explants.

Parameter	Healthy controls	Severe allergic asthmatics	p value
**N**	10	10	–
**Gender (M/F)**	3/7	3/7	–
**Age**	42.5 (30.75-52.25)	44 (38.5-52)	0.565
**Atopy (yes)**	0	10	–
**Total blood IgE**	16.45 (10.7-27.8)	172.7 (102.3-353)	0.0005***
**Blood lymphocytes**	1.9 (1.75-2.125)	1.9 (1.65-2.175)	0.413
**Blood eosinophils (%)**	0.1 (0.1-0.2)	0.2 (0.1-0.325)	0.105
**Pre FEV1**	3.11 (2.89-4.13)	2.57 (2.14-3.54)	0.044*
**Pre FEV1% predicted**	111.4 (105.3-117.1)	95.2 (74.95-104.7)	0.017*
**Post FEV1**	3.19 (3.04-4.19)	2.99 (2.37-3.69)	0.076
**Post FEV1% predicted**	114.1 (107.7-119.4)	100.6 (84.4-115.5)	0.073
**Sputum eosinophils (%)**	0	1.69 (0-3.68)	0.046*
**Sputum neutrophils (%)**	11.36 (7.125-15.72)	64.46 (21.91-74.2)	0.006**

Data shown are medians (inter-quartile range). Data were compared using Mann-Whitney U test. *p<0.05, **p<0.01, ***p<0.001.

## Methods

### Comparison of IMR expression on PBMCs at baseline in healthy and severe asthmatic participants

PBMCs were isolated from buffy coats by density centrifugation and 2 x 10^6^ PBMCs were used for IMR quantification by flow cytometry using a multiplexed panel of antibodies including CD14-AF488, CD56-PE/AF610, CD3-PE/Cy7, CD4-PerCP/Cy5.5 and CD8-APC in combination with antibodies directed against the IMRs (PD1, PDL-1, PDL-2, GITR, LIGHT, HVEM and BTLA) conjugated to the fluorophore phycoerythrin (PE). Additional samples were analysed for cell activation markers (HLA-DR-APC/Cy7 and CD25-BV421). Cells were analysed using a BD FACS ARIA flow cytometer and data analysed using proprietary FACS DIVA software.

### Sputum induction

Sputum induction and processing was performed as previously reported (13) at least 4 weeks after a respiratory tract infection. The mucous elements were selected from the expectorated sample and treated with 5 mM dithioerythritol in HEPES-buffered saline (HBS) to liquefy the mucus and obtain sputum cell cytospin slides as previously described (13). Differential cell counts were obtained using the rapid Romanowski method. Cell counts were expressed as the percentage of total respiratory cells (excluding squamous cell counts but including epithelial cells). A minimum of 6 weeks following the initial visit, participants were recalled, and 10 bronchial biopsies and 60 ml of blood were collected from each participant.

### Quantification of influenza infection, activation markers and IMR expression in resected lung tissue explants

Lung tissues were dissected into approximately 1 mm^3^ pieces and then rested overnight in 24-well culture dishes containing 500 μl of RPMI supplemented with glutamine and penicillin/streptomycin in a humidified tissue culture incubator at 37°C, 5% CO_2_. Explants were then cultured in RPMI medium supplemented with glutamine alone (RPMI-G) before adding the virus or virus diluent alone (0.4% w/v sucrose in 0.5 mM HEPES buffer, pH 7.4) and incubated for 2 h. Explants were then washed three times with basal RPMI medium to remove excess virus and incubated for 22 h in RPMI-G supplemented with 1% (w/v) BSA, after which conditioned media were centrifuged (400 g) to remove cellular material and stored at -80°C.

After adding influenza virus or carrier control, tissue explants were incubated for 24 h, following which cells within the tissues were enzymatically dispersed by vigorous agitation in collagenase type I diluted in culture medium for 60 min at 37°C. The dispersed cells were stained with BD fixable violet viability stain to identify dead cells, prior to further staining with two panels of antibodies directed against cell surface markers: one panel to identify T-lymphocyte subtypes including CD45-PE/CF594, CD3-PE/Cy7, CD8-APC, HLA-DR-APC/H7 and CD25-BV421, and the second to identify structural cells (Epithelial cells and fibroblasts) and macrophages using monoclonal antibodies specific for CD45-PE/CF594, HLA-DR-APC/Cy7,CD326-PerCP/Cy5.5 and CD90-APC.

The first panel was gated to identify CD8+ T cells (CD45+, CD3+, CD8+) and CD8- T cells (CD45+, CD3+, CD8-). The second panel was gated appropriately to identify alveolar macrophages (CD45+, CD3-, HLA-DR high), fibroblasts (CD45-, CD326-, CD90+) and epithelial cells (CD45-, CD90-, CD326+) (see **supplementary figure 1**). The stained dispersed cells were then split into equal portions and further stained with a panel of antibodies recognising IMRs (PD1, PDL-1, PDL-2, GITR, LIGHT, HVEM and BTLA) conjugated to the fluorophore phycoerythrin (PE). Samples stained for leukocytes were also used to quantify T-lymphocyte activation using the additional cell surface markers CD25-BV421 and HLA-DR-APC/Cy7. The dispersed cells were then fixed for 30 min with 2% paraformaldehyde to inactivate the influenza virus prior to analysis on the flow cytometer. Additional samples were fixed and permeabilised using a BD FixPerm kit prior to intracellular staining to quantify viral infection using a FITC-conjugated anti-viral nucleoprotein (NP) antibody (Abcam). This antibody recognises an intra-viral protein which is only present during replication and so distinguishes from the initial inoculum. Cells were analysed on a BD FACSAria equipped with three lasers (red, blue and violet), data were collected and analysed using BD FACS DIVA software. Infected tissues were compared to mock-infected (UV inactivated virus) controls using paired non-parametric tests.

### Comparison of the effects of ex-vivo influenza infection in bronchial explants

Up to 10 bronchial biopsies per participant were collected from ten healthy and ten severe asthmatic volunteers. After resting in in pairs in culture medium overnight, as previously described (14), explants were pre-treated for 2 h with 20 μg/ml control isotype antibody or anti-PD-1 blocking antibody in serum free culture medium. Explants were then rinsed with culture medium and blocking and isotype antibodies replenished prior to infection of the explants with log 7.4 pfu per well of A/H3N2/Wisconsin/67/2005 influenza virus stock solution. Explants were incubated for 2 h in the presence of the influenza virus/blocking Mab mix and were then rinsed thoroughly with serum-free culture medium prior to the addition of culture medium supplemented with 1% (w/v) human serum albumin and the blocking antibodies/isotype controls re-applied. Explants were then incubated for a further 22 h, after which conditioned supernatants were collected and centrifuged to remove contaminating cells prior to frozen storage at -80°C in aliquots.

Infected explants were carefully weighed and cells within tissues were dispersed as previously described (14) and stained with fixable violet viability stain to identify dead cells, prior to further staining with a panel of antibodies directed against cell surface markers as described for the resected lung tissue but using a simplified single panel due to small sample numbers. The panel consisted of: CD45-PE/CF594 (to differentiate leukocytes from structural cells), CD3-PE/Cy7, CD8-APC, HLA-DR-APC/H7, CD326-PerCP/Cy5.5, CD90-APC and CD25-BV421. The following cell populations were identified by sequential gating following exclusion of dead cells: CD8 T lymphocytes (CD45+, CD3+, CD8+), CD8- T lymphocytes (CD45+, CD3+, CD8-), Epithelial cells (CD45-, CD326+, CD90-) and fibroblasts (CD45-, CD326-, CD90+). The cells were then fixed and permeabilised using BD FixPerm kit prior to intracellular staining for influenza infection as before.

## Results and discussion

### IMR expression by peripheral blood cells in healthy and severe asthmatic participants

**Figure 1:**
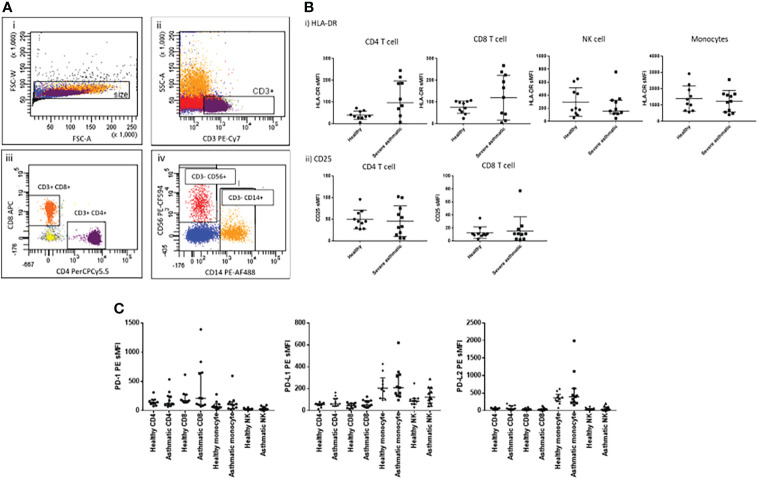
Peripheral immune cell phenotype in health and severe asthma. **(A)** Flow cytometric gating strategy for identification of immune cell sub-populations from freshly isolated PBMCs based on i) Size gate to exclude doublets, ii) gating on singlets, CD3+ T lymphocytes, iii) gating on CD3+ cells, CD4+ and CD8+ T lymphocytes, iv) gating on the CD3- population, CD56+ (NK cells) and CD14+ (monocytes). **(B)** Cell surface expression of activation markers in peripheral blood cell populations by flow cytometry, i) HLA-DR and ii) CD25. **(C)** PD-1, PD-L1 and PD-L2 cell surface expression on 4 key immune cell types in peripheral blood cells of healthy and severe asthmatic participants. N=10 per group, data were compared by Mann-Whitney tests.

While our primary aim was to examine the effect of influenza infection on localised lung tissue IMR responses in severe asthma, we needed first to determine the peripheral blood expression of IMRs at baseline in both healthy and severe asthmatic participants. We used multiplexed flow cytometry to examine first the activation status and IMR expression in different peripheral blood cell populations of 10 healthy non-asthmatic and 10 severe allergic asthmatic individuals (**Table 1**), identifying the blood cell sub-populations using flow cytometry (**Figure 1A**).

We found no significant alteration in peripheral T cell expression of either HLA-DR or CD25 in severe asthma (**Figure 1B**), although there was greater variance in HLA-DR expression in the asthmatic group, which could indicate a sub-group of asthmatics with some evidence of peripheral T cell activation. There was also no alteration in PD family member expression (**Figure 1C**) or in the wider IMR family members we examined (**supplementary Figure S1**). The mechanisms underlying the greater variance observed in HLA-DR and IMR expression in the asthmatic cohort are unclear, but could reflect as-yet undefined inflammatory cell phenotype changes in response to severe asthma. To limit the impact of current infections on T cell phenotype, blood samples were taken a minimum of four weeks post-exacerbation.

### Effect of influenza infection on IMR expression in peripheral lung tissues

In order to assess the effect of influenza infection on IMR expression in pulmonary tissues, peripheral lung tissue explants from 8 donors with median age 68 y (patient demographics summarised in **Table S1**) were exposed to influenza virus ex-vivo for 24 h as surrogates for bronchial tissues which do not provide sufficient cells for such complex analysis.

**Figure 2:**
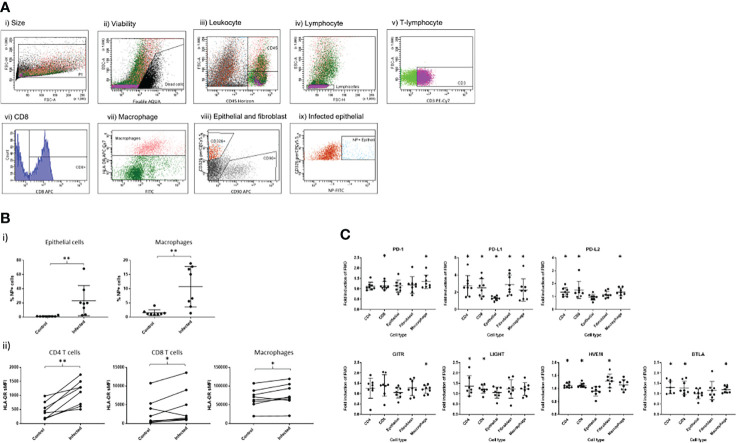
Effect of ex-vivo influenza infection on IMR expression in peripheral lung tissues. **(A)** Flow cytometric detection of dispersed cell populations from lung tissues post-exposure to influenza A virus. Dispersed cells were gated firstly on size to exclude doublets (i), then non-viable cells were excluded (ii). Leukocytes were identified using the CD45 cell surface marker, and lymphocytes subsequently by forward and side scatter properties (iv) prior to identification of T lymphocytes using CD3-PE/Cy7 (v). CD8-APC then identified CD8+ T cells (vi). CD45+/CD3- cell populations were used to gate for HLA-DR+ macrophages (vii). The CD45- cell population was used to identify epithelial and fibroblast cells using CD326-PerCP/Cy5.5 and CD90-APC respectively (viii). Infected epithelial cells were gated on CD45-/CD326+ cell population using NP-FITC, gating on mock-infected cells as a negative control. Data shown are representative of eight separate experiments. **(B)** i) Influenza infection measured by intracellular viral nucleoprotein (NP) expression in epithelial and macrophage cells dispersed from the infected lung tissues. ii) Effect of influenza infection on HLA-DR expression in CD8-, CD8+ T lymphocytes and macrophages. **(C)** Effect of influenza virus infection on fold change in expression of i) PD1 family immuno-modulatory proteins and ii) other IMRs by various cell populations measured as cell surface fluorescence-minus-one (FMO) staining intensity using flow cytometry. Asterisks indicate a significant fold change of expression following infection compared to non-infected controls (p<0.05). N=8, graphs show median +/- IQR, treatments were compared using Wilcoxon tests. *p<0.05, **p<0.01.

Epithelial cells, macrophages, CD4+ and CD8+ T cells were identified from the dispersed cell mixture using a flow cytometry gating strategy similar to that previously described (11) based on cell surface marker expression (**Figure 2A**). Infected cells were quantified by measurement of viral NP FITC fluorescence in the epithelial cell population. Median infection rates were 20% for epithelial cells and 10% for macrophages (**Figure 2B**i). We observed activation of tissue-resident T cells during infection in the same samples, with both CD4+ and CD8+ T cells exhibiting HLA-DR upregulation. Similar upregulation was seen on macrophages (**Figure 2B**ii), a process likely to be driven by mediator secretion as well as activation of innate intracellular anti-viral defences. Most of the measured mediators were elevated in conditioned media by *ex vivo* infection when compared to mock-infected control explants (**Figure S2C**), in particular those associated with innate response to viral infection, including IFN-γ, IL-2 and IP-10. We have previously observed significant up-regulation of eight of these mediators in ex-vivo lung tissues (14), and in this study many infection-responsive additional mediators which had not previously been assessed were added; these may serve as useful biomarkers or pathway targets in future studies.

We also found significant elevation of PD-1 expression on CD8+ T cells following acute exposure to influenza virus (**Figure 2C**i), consistent with the concept that these cells were activated either directly by exposure to viral antigens or indirectly as a result of increases in infection-induced cytokine/chemokine responses. In addition, we observed significant elevation of IL-2 and Il-15 (**Figure S2C**), mediators known to elevate T cell PD-1 (15). There was a trend towards similar increases of PD-1 expression on CD4+ T cells (p=0.074) and also significant up-regulation of PD-1 expression on macrophages following influenza infection. PD-1 expression on macrophages has been well described in the context of pathogen infection (16–19) and is known to negatively correlate with phagocytic potency of Mycobacterium tuberculosis (Mtb)-infected macrophages. Blockade of PD-1 *in-vivo* increases macrophage phagocytosis in Mtb infection models. In our model, it is unclear whether direct infection of the macrophages by influenza infection, or bystander effects of infection induced cytokines are responsible for this increase.

We found elevation of PD-L1 by influenza infection in lung tissues in all cell types studied (**Figure 2C**), consistent with widespread inducibility of this ligand in different cell types, a process known to be induced by IFN-γ expression (20). We found PD-L2 upregulated only on T cells and macrophages and not epithelial or fibroblast cells, consistent with previous reports in mice (21) and humans (22). PDL-2 is known to be upregulated by IL-4 and is correlated with alternatively activated macrophage markers in mice (23).

### Comparison of viral susceptibility/response in bronchial tissue of healthy and severe asthmatic participants

**Figure 3:**
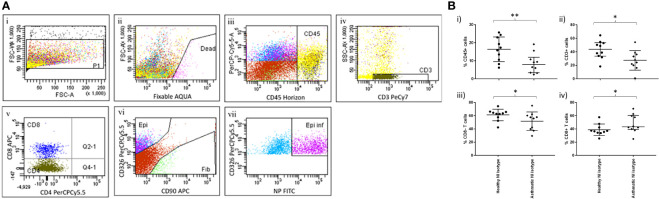
Immune cell identification and quantification in bronchial explants. Pairs of bronchial explants from n=10 healthy and asthmatic participants were enzymatically dispersed and the constituent cell populations were identified by flow cytometry. **(A)** Flow cytometric gating strategy to identify immune and structural cell populations. **(A)** i) Size gate to exclude doublets. ii) Viability gate to exclude dead cells. iii) Gating on live cells, identification of leukocytes using the CD45 marker. iv) Gating on CD45+ cells, identification of T lymphocytes using the CD3 marker. v) Gating on T lymphocytes, identification of CD8+ and CD8- T lymphocyte populations. vi) Gating on CD3- cells, identification of epithelial cells and fibroblasts using the CD326 and CD90 cell surface markers respectively. vii) Gating on epithelial cells, identification of infected cells using an intracellular stain for influenza virus nucleoprotein. Data are representative of 20 experiments. **(B)** Inflammatory cell load measured as a percentage of the parent cell populations in dispersed bronchial explants determined using the gating strategy shown previously, n=10 per group, bars are medians +/- IQR, NI=non-infected controls. Groups were compared using Mann-Whitney tests. *p<0.05, **p<0.01.

We then examined the effect of ex-vivo influenza infection on cells dispersed from bronchial biopsies from healthy and severe asthmatics. Cells dispersed from bronchial biopsies were identified using a gating strategy similar to that used in the peripheral lung tissue samples (**Figure 3A**). Because we had previously found insignificant levels of macrophages in bronchial tissue, and negligible infection rates in fibroblasts, we focused on epithelial cells, and CD8+/- T cells. Judging by numbers of dispersed cell populations, bronchial biopsies taken from severe asthma patients were relatively depleted in both overall leukocyte numbers, T lymphocytes and CD4+ T cells when measured as a percentage of all viable cells (**Figure 3B**), possibly due to treatment with inhaled steroids. Indeed, we have previously showed similar results in mild asthmatics who were taking lower overall doses of inhaled corticosteroids than in the current study (14). In contrast we found elevated CD8+T cell numbers in the current study, which could be explained by the severity of asthma in this group.

**Figure 4:**
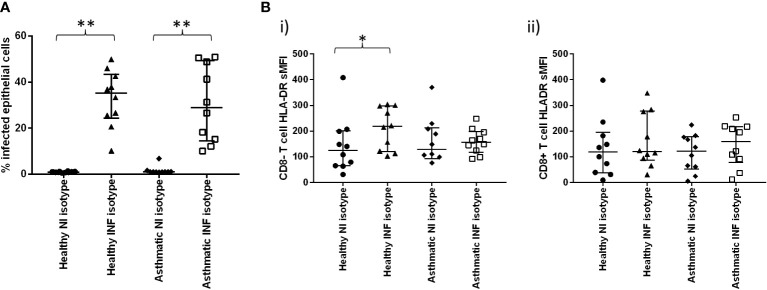
Effect of ex-vivo infection on T cell activation in bronchial biopsies. **(A)** Cells were dispersed from pair of bronchial biopsies 24-h post-infection with influenza virus and mock-infected controls. **(A)** Infected epithelial cells were measured by staining epithelial cells (CD45-, CD326+) for intracellular viral nucleoprotein using FITC-conjugated monoclonal antibodies and analysing by flow cytometry. **(B)** Cell surface expression of HLA-DR on CD8- and CD8+ T lymphocytes (CD45+/CD3+) post-infection, compared to mock-infected controls. n=10 per group, NI=non-infected controls, INF=influenza infected, bars are medians +/- IQR, Groups were compared using Mann-Whitney tests. *p<0.05, **p<0.01..

The infection rates were similar in healthy and asthmatic explants (**Figure 4A**), a somewhat surprising result considering the known susceptibility of asthmatics for viral infections but consistent with our previously reported study showing no differences in infection rates between healthy and mild asthmatic participants (24).

Infection was accompanied by marked activation of CD8- T cells (predominantly CD4+ T cells) but not CD8+ T cells (**Figure 4B**i, ii), and this effect was only observed in the healthy participants, suggesting aberrant activation signals in asthma, which could be due to treatment or inherent abnormalities in signalling pathways in this chronic inflammatory disease. Our previous work (24) suggests that, when steroid exposure is removed ex vivo, inflammatory suppression persists in asthmatic biopsies for at least 72 hours post-bronchoscopy.

Lack of CD8+ T cell activation in the bronchial compartment compared when compared to the resected lung tissue samples (see **Figure 1B**) could be due to the different organ location or different mediator accessibility in the bronchial tissues.

Ex-vivo data from epithelial cells and bronchoalveolar lavage fluid have previously indicated deficient type 1 interferon responses in asthmatics in response to viral challenge (25, 26). We found no evidence of deficient IFN-α2 following influenza challenge in the severe asthmatic group, and type II IFN levels (IFN-γ) were similar between the subject groups (**Figure S3A**). However, there was noticeable elevation of responses of several key T_H_2 cytokines in the asthmatics such as GRO, MDC and IL-13, as well as elevated IL-2, Il-1β, TNF-α and VEGF responses, either as primary or secondary responses to the Th2 cytokines. A number of these are associated with increased morbidity *in vivo*, and it is possible that these ex-vivo responses account for the increased morbidity and mortality seen in asthmatic patients during influenza infection.

**Figure 5:**
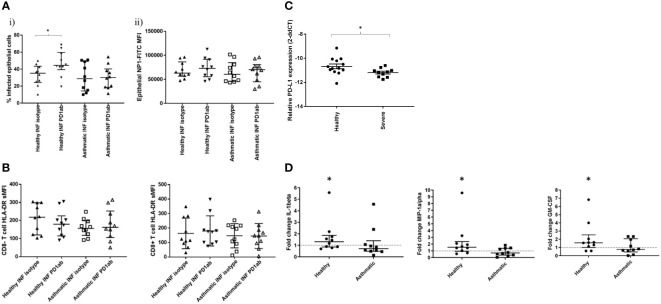
Effect of PD-1 blockade on influenza-induced T cell activation and inflammatory mediator production. Cells were dispersed from pair of bronchial biopsies 24-h post-infection with influenza virus.in the presence of PD1 blocking antibody or isotype controls. **(A)** Infected epithelial cells were measured by staining epithelial cells (CD45-, CD326+) for intracellular viral nucleoprotein using FITC-conjugated monoclonal antibodies and analysing by flow cytometry. **(B)** Cell surface expression of HLA-DR on CD8- and CD8+ T lymphocytes (CD45+/CD3+) dispersed from bronchial explants post-infection, compared to mock-infected controls. NI=non-infected controls, INF=influenza infected, Data shown are medians +/- IQR analysed by Wilcoxon test. **(C)** Comparison of PD-L1 RNA transcript levels in epithelial cell brushings measured by RT-PCR from healthy and severe asthmatic participants from an independent cohort. **(D)** Effect of PD-1 blockade on inflammatory mediator production by bronchial biopsies. Tissues were infected in the presence of PD-1 blocking monoclonal antibodies or isotype controls and incubated for a further 24 h in the presence of these antibodies. Tissue conditioned media were analysed for cytokines by ELISA. N=10 per group, data were compared using Wilcoxon matched pairs tests. *p<0.05 unadjusted p values. Line indicates the baseline fold change value of 1.

Intriguingly, inhibition of PD-1 signalling with blocking antibodies significantly increased the percentage of infected epithelial cells in the biopsies from healthy participants but not asthmatics (**Figure 5A**). To show that this was not an effect of increased intracellular production of the virus, we also measured intracellular antibody reactivity to the NP protein and found this to be unchanged, suggesting that increased numbers of infected cells, rather than increased intracellular viral load, were responsible for this change.

In the current study, we were unable to measure T cell activation using functional assays such as Elispot post-viral challenge, due to the very small numbers of T cells recoverable from the small biopsies used in the study, and, therefore, we had to rely on the T cell surface activation marker HLA-DR, as well as secreted mediator quantification, to examine the effect of checkpoint blockade on T cell activity. We found that PD-1 blockade had no significant effect on T cell activation (**Figure 5B**), suggesting that the blockade was working at activation levels below those that could be detected or through alternative cell types.

Examination of PD-L1 levels in epithelial cells of severe asthmatics compared to healthy control subjects in an independent cohort indicated a reduction in PD-L1 transcript levels in severe asthma (**Figure 5C**).

When mediator secretion in response to PD1 blockade was examined, blockade was only effective in altering the secretion of three mediators GM-CSF, IL-1B andMIP-1α (**Figure 5D** and **Figure S3B**), and then only in the healthy participants. In these cases, mediator secretion was increased, but only modestly, not by more than 2-fold.

PD-1 blockade did not alter IFN-γ as had been predicted in our initial assumption that CD8+ T cell activation would occur. Previous reports in chronic viral infections have shown both CD8+ T cell and CD4 T cell activation upon PD-1 blockade (27, 28). Recent evidence suggests that response to PD-1 blockade is complex and dependent upon the degree of T cell exhaustion and the complexity of the model system, since following chronic HCV exposure, combined CTLA and PD-1 blockade was necessary to restore T cell function, whereas individual PD-1 blockade was ineffective (29). Furthermore, there is some evidence that PD-1 blockade can be more effective in peripheral virus-specific T cells than in localised tissue virus-specific T cells, pointing to compartmentalisation of IMR blockade efficacy (30). The greater degree of co-receptor availability in complex ex-vivo tissue systems may have served to confound PD-1 blockade in our study. We demonstrated reduced PD-L1 transcript levels in epithelial cells from severe asthmatic subjects in an independent cohort, indicating the complex relationship between structural and immune cell IMR interactions likely to be present in tissue explants. There may also be additional redundancy in T cell regulation through more complex IMR induction patterns occurring as a result of infection in ex-vivo lung tissues, not just restricted to immune cell types, as we demonstrated in peripheral lung tissues (**Figure 2C**).

The pattern of response that we observed is suggestive that PD-1 blockade is mildly effective in promoting myeloid cell recruitment or activation post-infection and only in healthy people. In our previous work we have found that the deficit of cytokine production corresponds with increased susceptibility of bronchial tissues to influenza infection in asthma (24); however, PD-1 blockade did not significantly reduce the production of any of the cytokines measured in the current study (**Figure S3B**). These data, therefore, confirm that, whilst influenza infection appears to result in increased inflammatory mediator production in asthmatics, associated with Th2 phenotype, PD-1 blockade does not affect this pattern in any way. If anything, PD-1 blockade may be of more concern in healthy participants by enhancing myeloid cell activation or recruitment.

In summary, we have found that, unlike in COPD, there seems to be no difference between severe asthma and health in respect of IMR expression, at least in the systemic circulation. The study provides evidence of immune dysfunction in asthma compared to health, although due to a lack of direct measures of IMRs in bronchial tissue we could not determine if this was related to steroid treatment or IMRs. Finally, we have found that PD-1 blockade may be detrimental in healthy participants in the context of viral infection, which may have important implications for immunotherapy in patients who do not have any lung disease, and that IMR immunotherapy, although seemingly not beneficial for asthma itself, would not be detrimental in asthmatics requiring immunotherapy that targets IMRs for other conditions unrelated to asthma.

## Data availability statement

The datasets presented in this study can be found in online repositories. The names of the repository/repositories and accession number(s) can be found in the article/**Supplementary Material**.

## Ethics statement

The studies involving human participants were reviewed and approved by The study was approved by the regional Research Ethics Committee (REC), (Southampton and South West Hampshire Research Ethics Committee, LREC no: 09/H0504/109) and was conducted in accordance with Site Specific Assessment (SSA), and local Research and Development (R&D) approvals. The patients/participants provided their written informed consent to participate in this study.

## Author contributions

BN, MC and RD contributed to conception and design of the study. BN, JG, CB and HH-L contributed to experimental work. BN contributed to data analysis and statistics. BN and RD wrote the first draft of the manuscript. All authors contributed to the article and approved the submitted version.

## Acknowledgments

The authors would like to thank the Southampton NIHR respiratory biomedical research unit, the Southampton cardiothoracic surgical team, and the technical support staff at the Southampton Faculty of Medicine flow cytometry unit for their assistance with this work.

## Conflict of interest

The authors declare that the research was conducted in the absence of any commercial or financial relationships that could be construed as a potential conflict of interest.

This work was supported by a collaboration between the University of Southampton and Merck & Co Inc. Merck & Co Inc. assisted in the design of the work and the collection, analysis, and interpretation of data. The manuscript was conceived and written by the University of Southampton co-authors and was approved by co-authors from Merck & Co Inc.

## Publisher’s note

All claims expressed in this article are solely those of the authors and do not necessarily represent those of their affiliated organizations, or those of the publisher, the editors and the reviewers. Any product that may be evaluated in this article, or claim that may be made by its manufacturer, is not guaranteed or endorsed by the publisher.

## References

[1] Bardhan K Aksoylar HI Le Bourgeois T Strauss L Weaver JD Delcuze B. Phosphorylation of PD-1-Y248 is a marker of PD-1-mediated inhibitory function in human T cells. Sci Rep (2019) 9:17252. doi: 10.1038/s41598-019-53463-0 31754127 PMC6872651

[2] Wykes MN Lewin SR. Immune checkpoint blockade in infectious diseases. Nat Rev Immunol (2018) 18:91–104. doi: 10.1038/nri.2017.112 28990586 PMC5991909

[3] Jubel JM Barbati ZR Burger C Wirtz DC Schildberg FA. The role of PD-1 in acute and chronic infection. Front Immunol (2020) 11:487. doi: 10.3389/fimmu.2020.00487 32265932 PMC7105608

[4] Barber DL Wherry EJ Masopust D Zhu B Allison JP Sharpe AH. Restoring function in exhausted CD8 T cells during chronic viral infection. Nature (2006) 439:682–7. doi: 10.1038/nature04444 16382236

[5] McKendry RT Spalluto CM Burke H Nicholas B Cellura D Al-Shamkhani A. Dysregulation of antiviral function of CD8(+) T cells in the chronic obstructive pulmonary disease lung. role of the PD-1-PD-L1 axis. Am J Respir Crit Care Med (2016) 193:642–51. doi: 10.1164/rccm.201504-0782OC PMC4824936 26517304

[6] Van der Sluis RM Kumar NA Pascoe RD Zerbato JM Evans VA Dantanarayana AI. Combination immune checkpoint blockade to reverse HIV latency. J Immunol (2020) 204:1242–54. doi: 10.4049/jimmunol.1901191 PMC7354848 31988180

[7] Bengsch B Martin B Thimme R. Restoration of HBV-specific CD8+ T cell function by PD-1 blockade in inactive carrier patients is linked to T cell differentiation. J Hepatol (2014) 61:1212–9. doi: 10.1016/j.jhep.2014.07.005 25016223

[8] Raziorrouh B Kumar NA Pascoe RD Zerbato JM Evans VA Dantanarayana AI. Inhibitory phenotype of HBV-specific CD4+ T-cells is characterized by high PD-1 expression but absent coregulation of multiple inhibitory molecules. PloS One (2014) 9:e105703. doi: 10.1371/journal.pone.0105703 25144233 PMC4140833

[9] Nicholas B Bailey A Staples KJ Wilkinson T Elliott T Skipp P. Immunopeptidomic analysis of influenza a virus infected human tissues identifies internal proteins as a rich source of HLA ligands. PloS Pathog (2022) 18:e1009894. doi: 10.1371/journal.ppat.1009894 35051231 PMC8806059

[10] Kolle J Haag P Vuorinen T Alexander K Rauh M Zimmermann T. Respiratory infections regulated blood cells IFN-beta-PD-L1 pathway in pediatric asthma. Immun Inflammation Dis (2020) 8:310–9. doi: 10.1002/iid3.307 PMC7416032 32394602

[11] Bratke K Fritz L Nokodian F Geissler K Garbe K Lommatzsch M. Differential regulation of PD-1 and its ligands in allergic asthma. Clin Exp Allergy (2017) 47:1417–25. doi: 10.1111/cea.13017 28865147

[12] British Thoracic Society. Scottish Intercollegiate guidelines, n. British guideline on the management of asthma. Thorax (2008) 63:iv1–121. doi: 10.1136/thx.2008.097741 18463203

[13] Nicholas BL Skipp P Barton S Singh D Bagmane D Mould R. Identification of lipocalin and apolipoprotein A1 as biomarkers of chronic obstructive pulmonary disease. Am J Respir Crit Care Med (2010) 181:1049–60. doi: 10.1164/rccm.200906-0857OC PMC2874448 20110559

[14] Nicholas B Staples KJ Moese S Meldrum E Ward J Dennison P. A novel lung explant model for the ex vivo study of efficacy and mechanisms of anti-influenza drugs. J Immunol (2015) 194:6144–54. doi: 10.4049/jimmunol.1402283 PMC4456633 25934861

[15] Francisco LM Sage PT Sharpe AH. The PD-1 pathway in tolerance and autoimmunity. Immunol Rev (2010) 236:219–42. doi: 10.1111/j.1600-065X.2010.00923.x PMC2919275 20636820

[16] Huang X Venet F Wang YL Lepape A Yuan Z Chen Y. PD-1 expression by macrophages plays a pathologic role in altering microbial clearance and the innate inflammatory response to sepsis. Proc Natl Acad Sci U.S.A. (2009) 106:6303–8. doi: 10.1073/pnas.0809422106 PMC2669369 19332785

[17] Bally AP Lu P Tang Y Austin JW Scharer CD Ahmed R. NF-kappaB regulates PD-1 expression in macrophages. J Immunol (2015) 194:4545–54. doi: 10.4049/jimmunol.1402550 PMC4402259 25810391

[18] Chen W Wang J Jia L Liu J Tian Y. Attenuation of the programmed cell death-1 pathway increases the M1 polarization of macrophages induced by zymosan. Cell Death Dis (2016) 7:e2115. doi: 10.1038/cddis.2016.33 26913605 PMC4849159

[19] Shen L Gao Y Liu Y Zhang B Liu Q Wu J. PD-1/PD-L pathway inhibits m.tb-specific CD4(+) T-cell functions and phagocytosis of macrophages in active tuberculosis. Sci Rep (2016) 6:38362. doi: 10.1038/srep38362 27924827 PMC5141449

[20] Stanciu LA Bellettato CM Laza-Stanca V Coyle AJ Papi A Johnston SL. Expression of programmed death-1 ligand (PD-l) 1, PD-L2, B7-H3, and inducible costimulator ligand on human respiratory tract epithelial cells and regulation by respiratory syncytial virus and type 1 and 2 cytokines. J Infect Dis (2006) 193:404–12. doi: 10.1086/499275 16388488

[21] Yamazaki T Akiba H Iwai H Matsuda H Aoki M Tanno Y. Expression of programmed death 1 ligands by murine T cells and APC. J Immunol (2002) 169:5538–45. doi: 10.4049/jimmunol.169.10.5538 12421930

[22] Rodriguez-Garcia M Porichis F Jong OG Levi K Diefenbach TJ Lifson JD. Expression of PD-L1 and PD-L2 on human macrophages is up-regulated by HIV-1 and differentially modulated by IL-10. J Leukoc Biol (2011) 89:507–15. doi: 10.1189/jlb.0610327 PMC3058820 21097698

[23] Huber S Hoffmann R Muskens F Voehringer D. Alternatively activated macrophages inhibit T-cell proliferation by Stat6-dependent expression of PD-L2. Blood (2010) 116:3311–20. doi: 10.1182/blood-2010-02-271981 20625006

[24] Nicholas B Dudley S Tariq K Howarth P Lunn K Pink S. Susceptibility to influenza virus infection of bronchial biopsies in asthma. J Allergy Clin Immunol (2017) 140:309–312.e304. doi: 10.1016/j.jaci.2016.12.964 28259448

[25] Edwards MR Regamey N Vareille M Kieninger E Gupta A Shoemark A. Impaired innate interferon induction in severe therapy resistant atopic asthmatic children. Mucosal Immunol (2013) 6:797–806. doi: 10.1038/mi.2012.118 23212197 PMC3684776

[26] Sykes A Edwards MR Macintyre J del Rosario A Bakhsoliani E Trujillo-Torralbo MB. Rhinovirus 16-induced IFN-alpha and IFN-beta are deficient in bronchoalveolar lavage cells in asthmatic patients. J Allergy Clin Immunol (2012) 129:1506–1514.e1506. doi: 10.1016/j.jaci.2012.03.044 22657407

[27] Fisicaro P Valdatta C Massari M Loggi E Biasini E Sacchelli L. Antiviral intrahepatic T-cell responses can be restored by blocking programmed death-1 pathway in chronic hepatitis b. Gastroenterology (2010) 138:682–693, 693.e681-684. doi: 10.1053/j.gastro.2009.09.052 19800335

[28] Tang ZS Hao YH Zhang EJ Xu CL Zhou Y Zheng X. CD28 family of receptors on T cells in chronic HBV infection: Expression characteristics, clinical significance and correlations with PD-1 blockade. Mol Med Rep (2016) 14:1107–16. doi: 10.3892/mmr.2016.5396 PMC4940091 27314219

[29] Nakamoto N Cho H Shaked A Olthoff K Valiga ME Kaminski M. Synergistic reversal of intrahepatic HCV-specific CD8 T cell exhaustion by combined PD-1/CTLA-4 blockade. PloS Pathog (2009) 5:e1000313. doi: 10.1371/journal.ppat.1000313 19247441 PMC2642724

[30] Nakamoto N Kaplan DE Coleclough J Li Y Valiga ME Kaminski M. Functional restoration of HCV-specific CD8 T cells by PD-1 blockade is defined by PD-1 expression and compartmentalization. Gastroenterology (2008) 134:1927–1937, 1937.e1921-1922. doi: 10.1053/j.gastro.2008.02.033 18549878 PMC2665722

