# Supplementary material for: Immunomodulatory regulator blockade in a viral exacerbation model of severe asthma
Source: Front Immunol. 2022 Nov 21;13:973673. doi: 10.3389/fimmu.2022.973673 (PMC9720166; doi:10.3389/fimmu.2022.973673)
Supplement: Supplementary file 1 [file Presentation_1.pptx]

## Slide 1
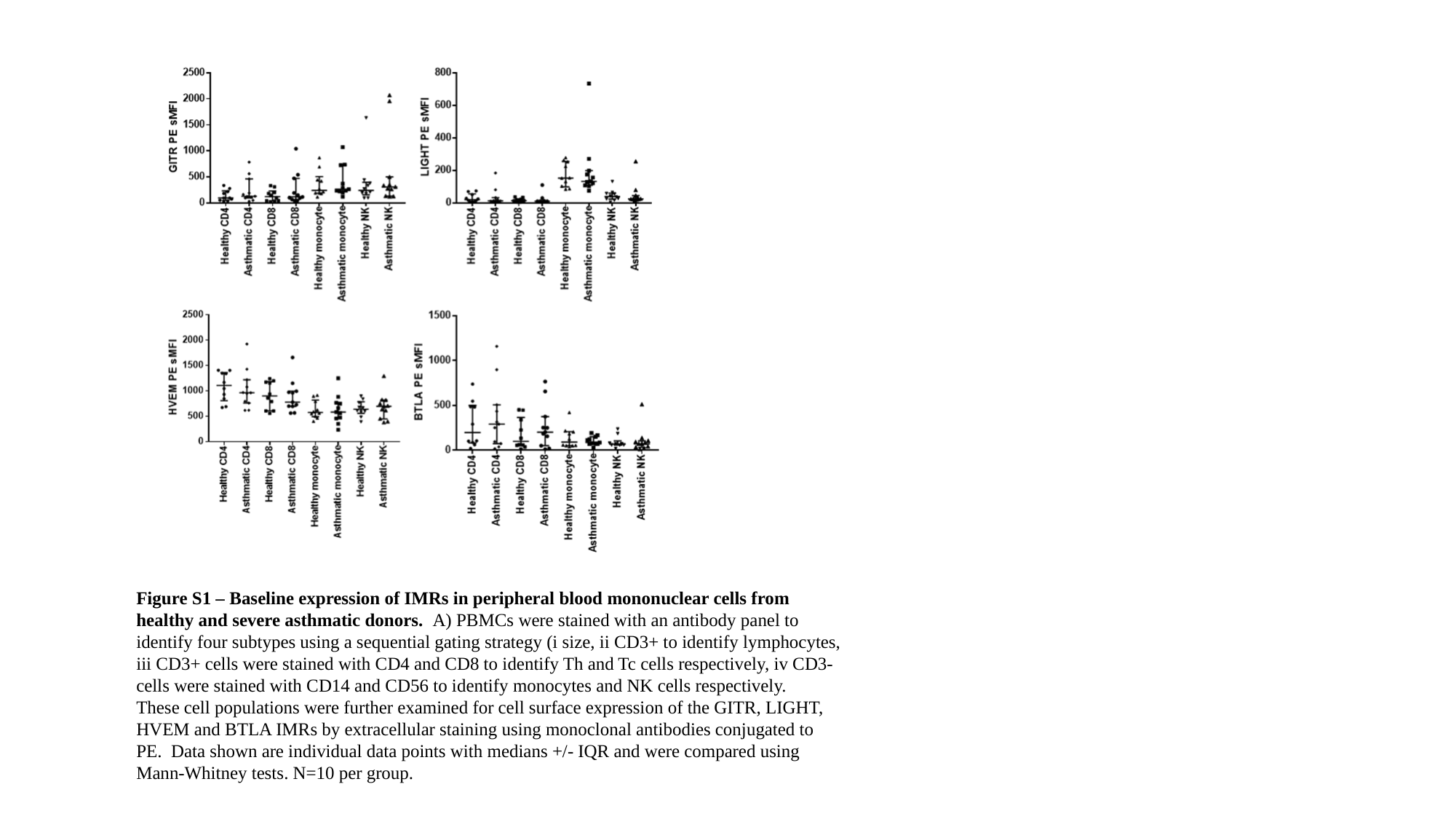

Figure S1 – Baseline expression of IMRs in peripheral blood mononuclear cells from healthy and severe asthmatic donors. A) PBMCs were stained with an antibody panel to identify four subtypes using a sequential gating strategy (i size, ii CD3+ to identify lymphocytes, iii CD3+ cells were stained with CD4 and CD8 to identify Th and Tc cells respectively, iv CD3- cells were stained with CD14 and CD56 to identify monocytes and NK cells respectively. These cell populations were further examined for cell surface expression of the GITR, LIGHT, HVEM and BTLA IMRs by extracellular staining using monoclonal antibodies conjugated to PE. Data shown are individual data points with medians +/- IQR and were compared using Mann-Whitney tests. N=10 per group.
