# Supplementary material for: Immunomodulatory regulator blockade in a viral exacerbation model of severe asthma
Source: Front Immunol. 2022 Nov 21;13:973673. doi: 10.3389/fimmu.2022.973673 (PMC9720166; doi:10.3389/fimmu.2022.973673)
Supplement: Supplementary file 2 [file Presentation_2.pptx]

## Slide 1
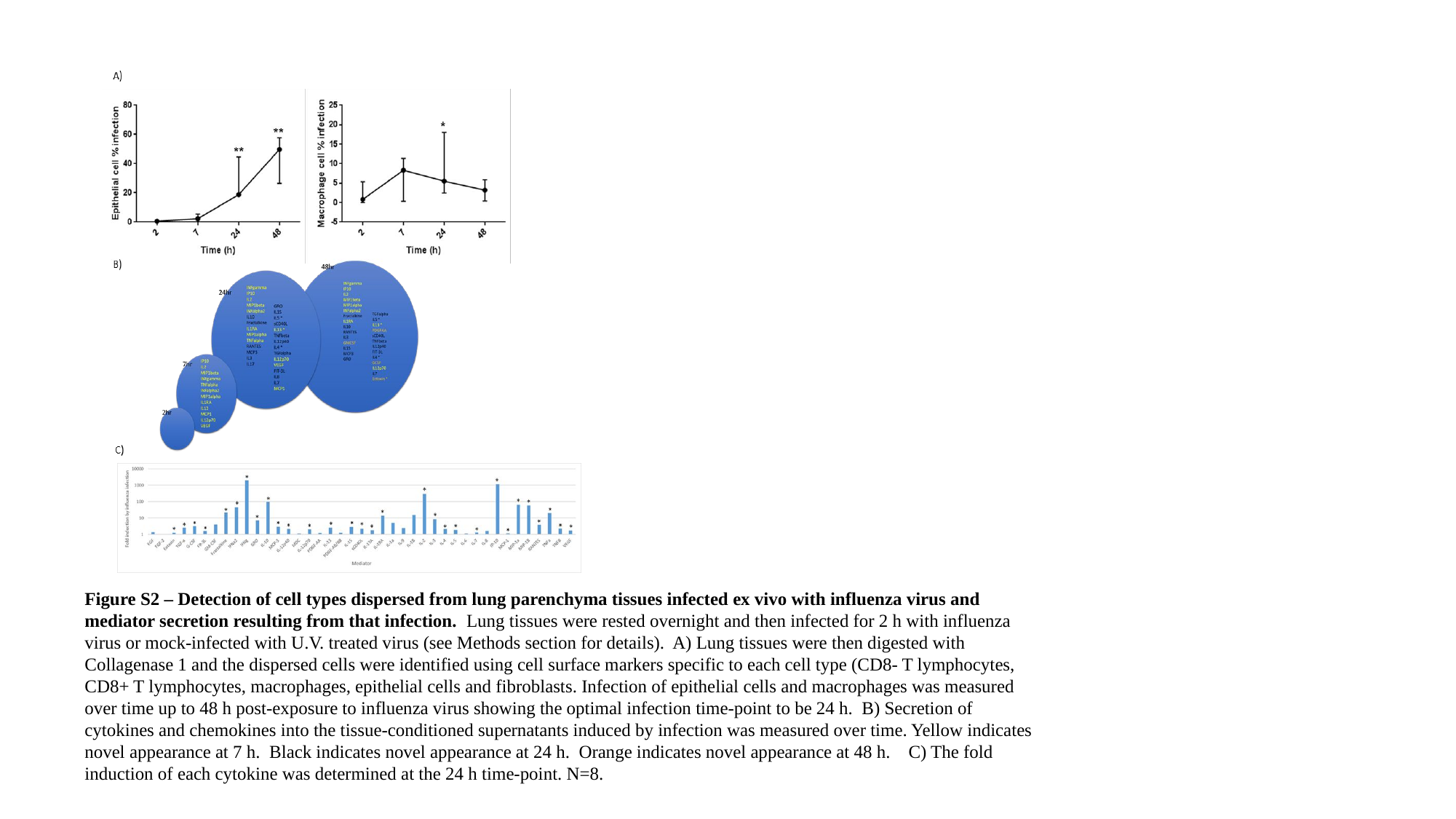

Figure S2 – Detection of cell types dispersed from lung parenchyma tissues infected ex vivo with influenza virus and mediator secretion resulting from that infection. Lung tissues were rested overnight and then infected for 2 h with influenza virus or mock-infected with U.V. treated virus (see Methods section for details). A) Lung tissues were then digested with Collagenase 1 and the dispersed cells were identified using cell surface markers specific to each cell type (CD8- T lymphocytes, CD8+ T lymphocytes, macrophages, epithelial cells and fibroblasts. Infection of epithelial cells and macrophages was measured over time up to 48 h post-exposure to influenza virus showing the optimal infection time-point to be 24 h. B) Secretion of cytokines and chemokines into the tissue-conditioned supernatants induced by infection was measured over time. Yellow indicates novel appearance at 7 h. Black indicates novel appearance at 24 h. Orange indicates novel appearance at 48 h. C) The fold induction of each cytokine was determined at the 24 h time-point. N=8.
