# Supplementary material for: Immunomodulatory regulator blockade in a viral exacerbation model of severe asthma
Source: Front Immunol. 2022 Nov 21;13:973673. doi: 10.3389/fimmu.2022.973673 (PMC9720166; doi:10.3389/fimmu.2022.973673)
Supplement: Supplementary file 3 [file Presentation_3.pptx]

## Slide 1
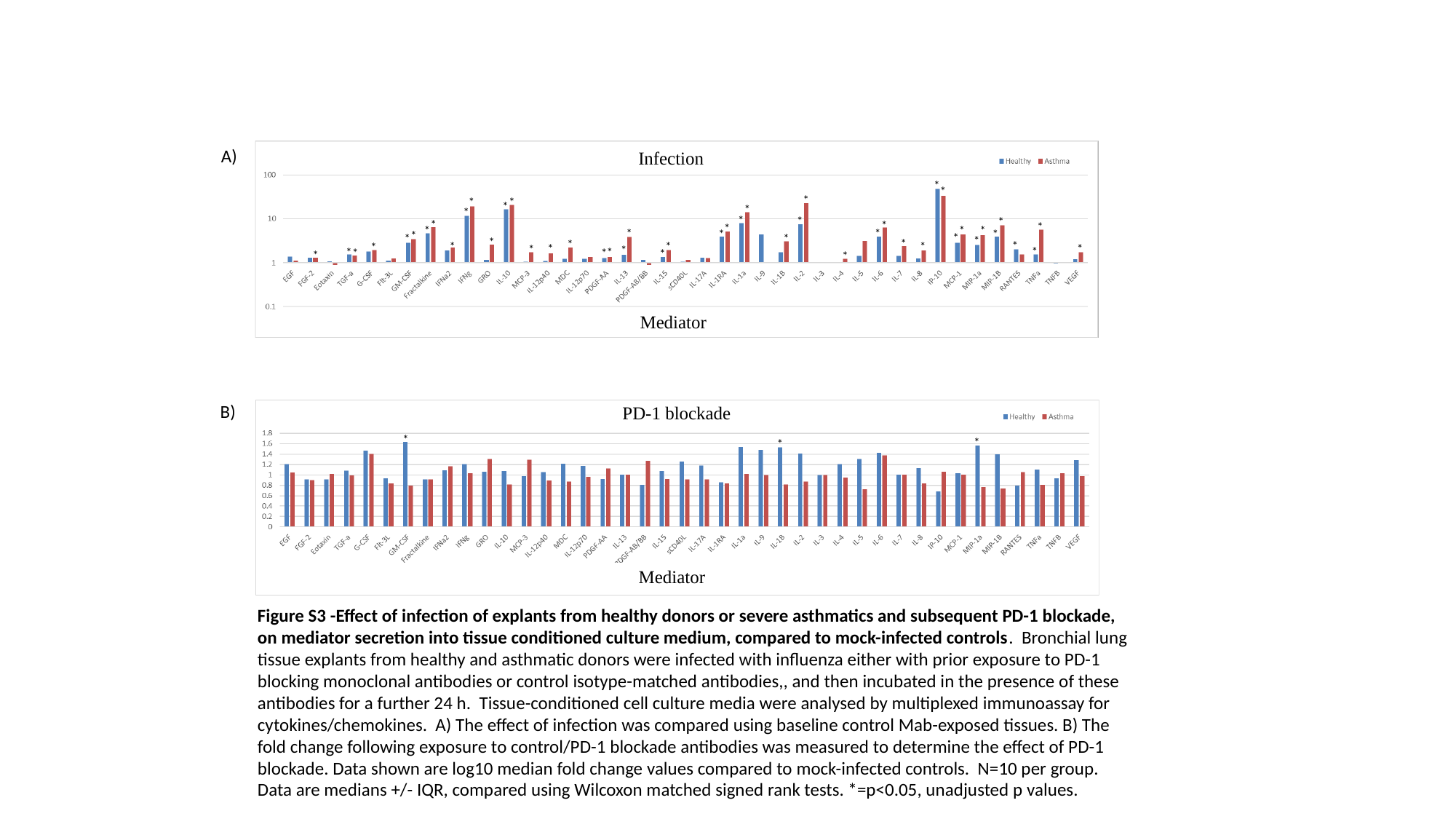

A)
Infection
*
*
*
*
*
*
*
*
*
*
*
*
*
*
*
*
*
*
*
*
*
*
*
*
*
*
*
*
*
*
*
*
*
*
*
*
*
*
*
*
*
*
*
*
*
*
*
Mediator
B)
PD-1 blockade
*
*
*
Mediator
Figure S3 -Effect of infection of explants from healthy donors or severe asthmatics and subsequent PD-1 blockade, on mediator secretion into tissue conditioned culture medium, compared to mock-infected controls. Bronchial lung tissue explants from healthy and asthmatic donors were infected with influenza either with prior exposure to PD-1 blocking monoclonal antibodies or control isotype-matched antibodies,, and then incubated in the presence of these antibodies for a further 24 h. Tissue-conditioned cell culture media were analysed by multiplexed immunoassay for cytokines/chemokines. A) The effect of infection was compared using baseline control Mab-exposed tissues. B) The fold change following exposure to control/PD-1 blockade antibodies was measured to determine the effect of PD-1 blockade. Data shown are log10 median fold change values compared to mock-infected controls. N=10 per group. Data are medians +/- IQR, compared using Wilcoxon matched signed rank tests. *=p<0.05, unadjusted p values.
