# Supplementary material for: Immunomodulatory regulator blockade in a viral exacerbation model of severe asthma
Source: Front Immunol. 2022 Nov 21;13:973673. doi: 10.3389/fimmu.2022.973673 (PMC9720166; doi:10.3389/fimmu.2022.973673)
Supplement: Supplementary file 4 [file Table_1.docx]

**Supplementary information**

**Table S1** – Demographics of volunteers donating resected peripheral lung tissue samples used for initial assessment of IMR expression and ex vivo responses to influenza infection.

|  | **Resected tissue donors** |
| --- | --- |
| **n** | 8 |
| **M/F** | 4/4 |
| **Age** | 68.5 |
| **Diagnosis** | 5 Adeno, 2 squamous, 1 Met |
| **Smoking status** | 5 Ex, 2 current, 1 NK |
